# Parkinsonian Syndromes in Motor Neuron Disease: A Clinical Study

**DOI:** 10.3389/fnagi.2022.917706

**Published:** 2022-06-27

**Authors:** Jacopo Pasquini, Francesca Trogu, Claudia Morelli, Barbara Poletti, Floriano Girotti, Silvia Peverelli, Alberto Brusati, Antonia Ratti, Andrea Ciammola, Vincenzo Silani, Nicola Ticozzi

**Affiliations:** ^1^Department of Neurology and Laboratory of Neuroscience, Istituto Auxologico Italiano IRCCS, Milan, Italy; ^2^Neurology Residency Program, Università Degli Studi di Milano, Milan, Italy; ^3^Clinical Ageing Research Unit, Newcastle University, Newcastle upon Tyne, United Kingdom; ^4^Department of Brain and Behavioral Sciences, Università degli Studi di Pavia, Pavia, Italy; ^5^Department of Medical Biotechnology and Translational Medicine, Università Degli Studi di Milano, Milan, Italy; ^6^Department of Pathophysiology and Transplantation, Dino Ferrari Center, Università Degli Studi di Milano, Milan, Italy

**Keywords:** motor neuron disease (MND), parkinsonism, amyotrophic lateral sclerosis, primary lateral sclerosis (PLS), progressive supranuclear palsy

## Abstract

**Background:**

Parkinsonian syndromes may rarely occur in motor neuron disease (MND). However, previous studies are heterogeneous and mostly case reports or small case series. Therefore, we aimed to identify and characterize patients with concurrent parkinsonian syndromes extracted from a cohort of 1,042 consecutive cases diagnosed with MND at a tertiary Italian Center.

**Methods:**

Diagnosis of Parkinson's disease (PD), progressive supranuclear palsy (PSP) and corticobasal syndrome (CBS) was made according to current criteria. Clinical characterization included: upper and lower motor neuron disease features, typical and atypical parkinsonian features, oculomotor disorders, cognitive testing, MRI features, and, when available molecular neuroimaging. Genetic testing was carried out for major MND and PD-associated genes.

**Results:**

Parkinsonian syndromes were diagnosed in 18/1042 (1.7%) of MND patients (7 PD, 6 PSP, 3 CBS, 2 other parkinsonisms). Based on phenotype, patients could be categorized into amyotrophic lateral sclerosis (ALS)-parkinsonism and primary lateral sclerosis (PLS)-parkinsonism clusters. Across the whole database, parkinsonism was significantly more common in PLS than in other MND phenotypes (12.1 vs. 1.1%, *p* = 5.0 × 10^−10^). MND patients with parkinsonian features had older age of onset, higher frequency of oculomotor disorders, cognitive impairment, and family history of parkinsonism or dementia. Two patients showed pathogenic mutations in *TARDBP* and *C9orf72* genes.

**Conclusion:**

Specific patterns in MND-parkinsonism were observed, with PLS patients often showing atypical parkinsonian syndromes and ALS patients more frequently showing typical PD. Systematic clinical, genetic, and neuropathologic characterization may provide a better understanding of these phenotypes.

## Introduction

Parkinsonian syndromes can occur in motor neuron diseases (MND), accompanying upper (UMN) and lower motor neuron (LMN) signs (amyotrophic lateral sclerosis, ALS-parkinsonism), UMN signs alone (primary lateral sclerosis, PLS-parkinsonism) and, less often, LMN signs alone (Qureshi et al., 1996; Sudo et al., 2002; Hideyama et al., 2006; Pradat et al., 2009; Gilbert et al., 2010; Calvo et al., 2019).

Some epidemiological studies showed a higher prevalence of Parkinson's disease (PD) among relatives of MND patients, suggesting a common clinicopathological spectrum rather than coincidental events (Eisen and Calne, 1992; Qureshi et al., 1996), and pathological evidence of nigrostriatal denervation was shown in MND-parkinsonism overlap syndromes (Sudo et al., 2002). The phenotypic variability of overlap syndromes is occasionally explained by the presence of pathogenic mutations in several genes, some recognized as causative genes for sporadic and familial ALS and Frontotemporal Dementia (FTD) such as *C9orf72* (Ticozzi et al., 2014). While the link between ALS and FTD has been well described in recent years, the focus of our study was to describe MND phenotypes with overlapping parkinsonian syndromes. Studies reporting parkinsonian syndromes overlapping MND are heterogeneous with regard to L-dopa responsiveness, molecular imaging evidence of nigrostriatal dysfunction, presence of cognitive impairment, and other additional features (e.g., oculomotor disorders) (Zoccolella et al., 2002; Mackenzie and Feldman, 2004; Pradat et al., 2009; Lim et al., 2013; Calvo et al., 2019). Therefore, the aims of this study were to: (i) report the frequency of parkinsonian syndromes in a large cohort of Italian MND patients that were evaluated at our tertiary ALS center; (ii) provide a clinical description and categorization of patients with MND and parkinsonian syndromes.

## Patients and Methods

### Study Design and Patient Selection

**Figure 1:**
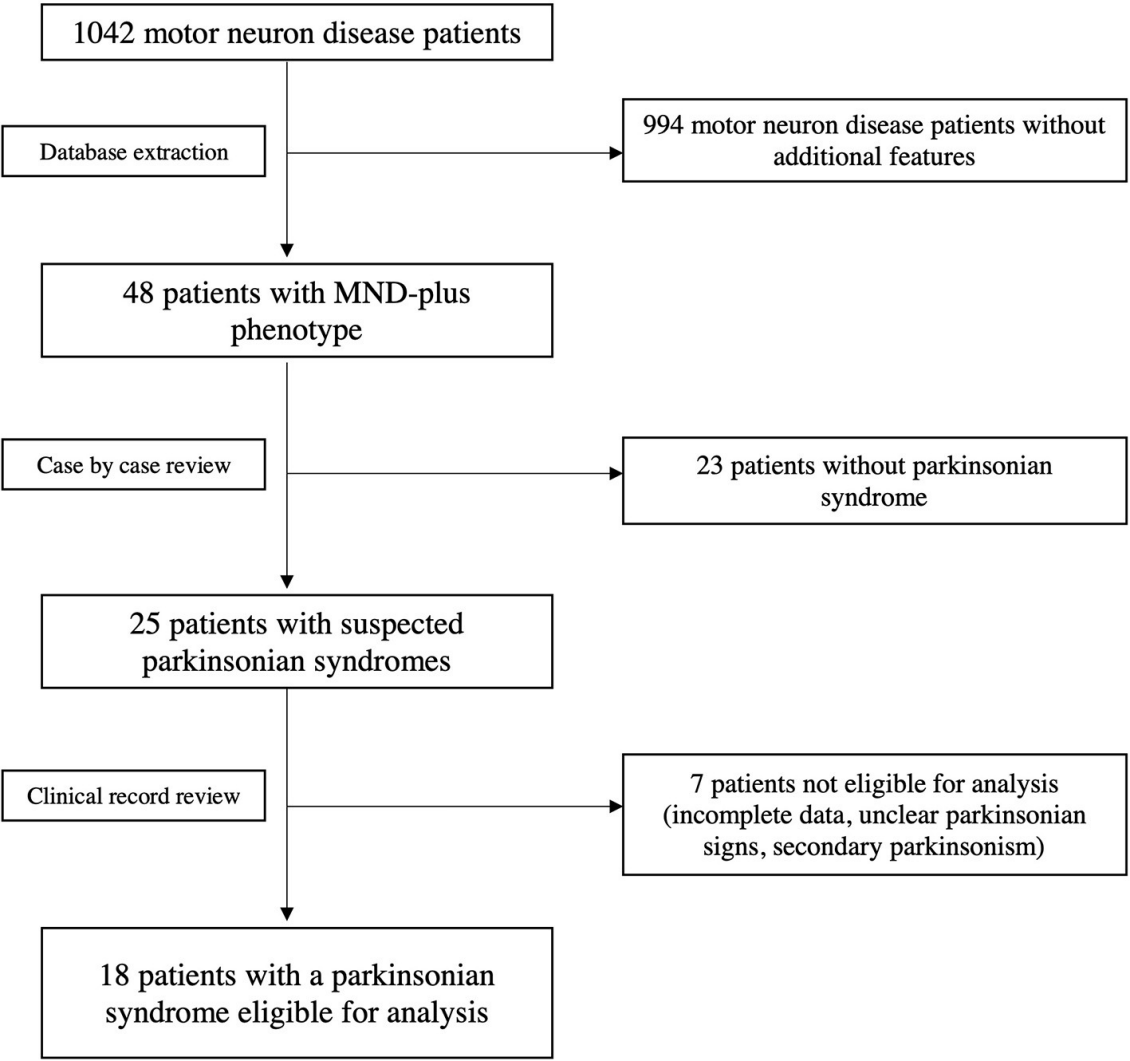
Flow-chart showing database extraction and selection of patients with overlapping MND and primary parkinsonian syndromes.

This single center observational study was conducted at the Department of Neurology, Istituto Auxologico Italiano IRCCS, a tertiary MND center. All patients with a clinical diagnosis of ALS and other MNDs according to the El Escorial revised criteria were consecutively recorded in an electronic database (Brooks et al., 2000). The database contains structured demographic and clinical information, which is manually entered from each patient's clinical record. The database was built on January 2008 and accessed on January 10^th^ 2022, when 1042 individual patients' records were present. Patients with a MND “plus” phenotype (defined as presence of prominent sensory, ocular, cerebellar, autonomic or parkinsonian sign and symptoms) were extracted, and those receiving a diagnosis of MND overlapping with parkinsonian syndromes [Parkinson's disease (PD), corticobasal syndrome (CBS), progressive supranuclear palsy (PSP) and multisystem atrophy (MSA)] at the time of evaluation were considered for this analysis (Figure 1). Causes of secondary parkinsonism were ruled out. Diagnoses of parkinsonism were formulated at the time of clinical evaluation. Based on the available criteria at the time of the evaluation, UK Parkinson's Disease Society (PDS) and Brain Bank criteria were used for the diagnosis of parkinsonian syndromes and idiopathic PD (Hughes et al., 1992). National Institute of Neurological Disorders and Stroke – Society for PSP (NINDS-SPSP) and Movement Disorders Society (MDS) criteria were considered when evaluating features of PSP (Litvan et al., 1996; Höglinger et al., 2017), while for CBS diagnosis the criteria proposed by Boeve and Armstrong were adopted (Boeve et al., 2003; Armstrong et al., 2013). Furthermore, to confirm the validity of the clinical data supporting the diagnosis, the original clinical records of the selected patients were also independently reviewed by clinicians experienced in the field of MND (NT) and movement disorders (JP).

### Parkinsonian Features

The following features were recorded at the time of the evaluation in all MND-parkinsonism patients: rigidity with a clear cogwheeling phenomenon; rest or re-emergent tremor; bradykinesia with a clear slowing and decrement of amplitude during finger tapping; gait: short and shuffling steps, freezing of gait, trunk anteroflexion or camptocormia not attributed to muscle weakness, reduced arm swing not attributed to spasticity; postural instability; ocular movement disorders. Response to levodopa, molecular imaging evidence of dopamine transporter deficiency in the striatum, and genetic screening of genes associated to parkinsonian syndromes were also recorded when available (SNCA, PRKN, PINK1, DJ1, LRRK2, ATP13A2, DNAJC6, FXBO7, GBA, PARK7, RAB39B, VPS13C, VPS35, PLA2G6, DCTN1, GCH1, GRN, MAPT, PRNP, PSEN1, PSEN2).

### Motor Neuron Disease Features

The following characteristics were considered in all MND patients: motor neuron disease phenotype, age and site of onset, revised ALS Functional Rating Scale (ALSFRS-r) (Cedarbaum, 1996), disease duration, cognitive and behavioral features evaluated at standard neuropsychological testing, presence of mutations in the *SOD1, TARDBP, FUS* and *C9orf72* genes. Additionally, when present, the following information were collected: genetic screening of other ALS-associated genes, brain MRI features, cerebrospinal fluid (CSF) analysis, other clinical and neuroimaging analyses (e.g. ^18^F-FDG PET), pre-existing medical conditions.

### Statistical Analysis

Comparisons between groups were performed with Mann-Whitney U test for quantitative variables and with chi-square (χ^2^) test for categorical variables. Survival analysis (tracheotomy or death) was performed with the Kaplan-Meier method. The significance threshold for hypothesis testing was *p* < 0.05. Statistical analysis was carried out with Statistical Package for Social Sciences (IBM® SPSS) version 26. Pairwise deletion was used to handle missing data.

### Ethical Standards and Data Availability

This study was approved by the ethical committee of Istituto Auxologico Italiano IRCCS (project DAMARE) and was performed in accordance with the 1964 Declaration of Helsinki and its later amendments. Written informed consent for using anonymized clinical data for research purposes was obtained at the time of evaluation from all participants. Pseudo-anonymized datasets analyzed for this study are archived on Zenodo (doi: 10.5281/zenodo.5647409) and will be shared upon reasonable request.

## Results

We identified in our cohort 18/1042 (1.7%, 8 female and 10 male) patients with MND-parkinsonism. Among these, seven patients (0.7%) received a diagnosis of concurrent PD, six of PSP (0.6%), three of CBS (0.3%). Two (0.2%) patients showed a progressive, akinetic-rigid, primary parkinsonian syndrome not responsive to levodopa treatment, but without elements suggestive of atypical parkinsonisms. No case of concurrent MSA and MND was observed. Based on the pattern of UMN and LMN signs, as well as parkinsonian features, MND-parkinsonism patients were grouped into two main phenotypic clusters. The first cluster was composed of 11 patients with ALS and parkinsonism: among these, seven patients had concurrent idiopathic PD, two received a diagnosis of CBS, while the remaining two manifested overt bradykinesia associated to cogwheel rigidity and/or rest tremor, not responsive to levodopa therapy, but without other signs or symptoms suggestive for atypical parkinsonian syndromes. Within this cluster, most patients (7/11) displayed a MND phenotype with prominent LMN signs (classic ALS, flail arm or flail leg syndromes), while bulbar and pyramidal ALS were each observed in two patients. The second phenotypic cluster includes seven patients with PLS and atypical parkinsonian syndromes characterized by axial rigidity, postural instability and oculomotor dysfunction (mainly slow saccades, vertical gaze palsy and ocular apraxia). Within this group, six individuals met the criteria for a diagnosis of concurrent PSP, while the remaining one had signs suggestive of CBS. Disease onset was in the bulbar segment in four patients and in the spinal muscles in the remaining three cases. Interestingly, in 5/7 patients with ALS and idiopathic PD, the PD diagnosis preceded the MND onset by several months or years. Conversely, in all PLS patients, atypical parkinsonism was present at the time of diagnosis or appeared later in the disease course. Demographic and clinical characteristics of patients with MND-parkinsonism are summarized in Table 1 and Supplemental Table 1.

**Table 1:** Detailed clinical characteristic of the 18 patients with a MND-parkinsonism phenotype.

**ID**	**MND onset (y)**	**Park onset** **(y)**	**Sex**	**MND clinical phenotype**	**MND onset (site)**	**Time at first visit**	**Survival (months)**	**Parkinsonism clinical phenotype**	**Rigidity**	**Tremor**	**Brady kinesia**	**Posture** **and gait**	**Arm swing**	**Postural instability**	**Eye movements**	**Other**	**LD response**	**Cognitive decline**	**DaT imaging**
**1**	55.2	58.8	F	PLS	B	27.3	78.5†	PSP	UL asymmetric	N	Y	Freezing and festination	N	Y	Fragmented smooth pursuit	Hyposmia; hypomimia	Y	Mild memory impairment	NA
**2**	49.1	49.2	M	PLS	LL	18.0	35.3	CBS	UL asymmetric	N	Y	Camptocormia	N	Y	N	Left hand and foot dystonia, hypomimia	N	Frontal cognitive decline, left limb apraxia	Normal
**3**	61.4	63.0	F	PLS	B	19.1	47.1	PSP	UL asymmetric	Postural tremor	N	N	N	N	Upgaze and lateral limitation	Hypomimia	NA	Frontal cognitive decline	NA
**4**	77.6	77.6	F	PLS	LL	12.2	36.0†	PSP	UL asymmetric	N	N	Unstable	Reduced	Y	Upgaze limitation; slow saccades	N	NA	Frontal cognitive decline	NA
**5**	76.4	76.4	M	PLS	B	28.8	76.9†	PSP	UL asymmetric	Rest tremor	Y	Wide based	Reduced	Y	Vertical and horizontal gaze limitation; slow saccades; fragmented smooth pursuit	N	NA	Frontal cognitive decline	NA
**6**	43.2	43.3	M	PLS	UL	8.4	25.4†	PSP	UL symmetric	N	Y	Trunk anteroflexion	Reduced	N	Very slow saccades; gaze apraxia	Hypomimia	Poor	Frontal dysexecutive syndrome, apathy	Bilateral asymmetric reduction
**7**	66.6	69.6	F	PLS	B	12.4	102.7†	PSP	UL asymmetric	N	Y	N	N	Y	Slow horizontal saccades; upgaze limitation	Hypomimia	Y	Frontal cognitive decline	Baseline: normal, 5y FU: mild reduction right putamen
**8**	80.1	80.1	M	ALS-classic	LL	18.0	38.0	PD	UL and LL contralaterally	N	Y	Trunk anteroflexion	Reduced	N	N	Hypomimia	Y	Subclinical frontal cognitive impairment	NA
**9**	70.7	71.0	M	ALS-classic	UL	6.4	18.3	PD	All limbs asymmetric	N	Y	Trunk anteroflexion; festination	N	N	Fragmented smooth pursuit	N	Y	Subclinical frontal cognitive impairment	Bilateral asymmetric reduction
**10**	68.1	62.0	M	ALS-classic	LL	5.8	15.9	PD	UL asymmetric	Rest tremor	Y	Trunk anteroflexion	Reduced	Y	Fragmented smooth pursuit; upgaze limitation; downward slow saccades	Hypomimia	Y	N	NA
**11**	68.3	67.9	M	ALS-flail leg	LL	39.9	61.0†	PD	UL asymmetric	Rest tremor	Y	Unable to stand	Unable to stand	Unable to stand	N	N	Partial	N	Bilateral asymmetric reduction
**12**	64.2	61.1	M	ALS-flail arm	UL	12.4	17.9†	PD	All limbs asymmetric	Rest remor	Y	Trunk anteroflexion	N	N	N	N	Y	N	Abnormal
**13**	78.7	77.6	M	ALS-bulbar	B	9.8	36.6	PD	UL asymmetric	Rest tremor, asymmetric; kinetic tremor	Y	N	Reduced	Y	Upgaze limitation	Micrographia, hypomimia	Y	N	NA
**14**	67.2	64.4	F	ALS-classic	LL	10.7	10.7†	PD	UL asymmetric	Postural and kinetic	Y	Unable to stand unaided	Unable to stand unaided	Unable to stand unaided	N	Hypomimia	Partial	Mild memory deficit	Bilateral asymmetric reduction
**15**	60.8	66.5	M	ALS-bulbar	B	45.9	104.3†	Parkinsonism	UL symmetric	N	Y	N	Reduced	Y	Fragmented smooth pursuit; slow horizontal saccades.	N	N	Mild memory and frontal deficits	NA
**16**	75.9	75.9	F	ALS-UMNp	LL	4.7	6.7	Parkinsonism	All limbs asymmetric	N	Y	N	N	N	Fragmented smooth pursuit; slow saccades	N	N	N	NA
**17**	61.8	61.9	F	ALS-UMNp	UL	7.4	25.3	CBS	All limbs	Intermittent right hand rest tremor	Y	N	Reduced	N	Slow saccades in all directions	Hypomimia; left hand dystonia and apraxia	N	Mild memory deficit and frontal dysexecutive syndrome buccofacial apraxia	Normal
**18**	65.7	65.1	F	ALS-classic	UL	24.6	62.4	CBS	LL asymmetric	N	Y	Trunk anteroflexion; festination	Reduced	Y	Ocular apraxia	Hypomimia, left hand apraxia	Partial	Frontal cognitive decline, ideomotor and buccofacial apraxia	Bilateral asymmetric reduction

*The table has been subdivided in two parts according to the following grouping: PLS-parkinsonism (IDs 1-7) and ALS-parkinsonism (IDs 8-18). Rigidity was considered to have parkinsonian characteristics when showing a clear cogwheeling phenomenon. Tremor was considered to have parkinsonian characteristics when showing a clear rest component. The presence of postural and kinetic is also reported. Bradykinesia was considered to have parkinsonian characteristics when showing a clear slowing and reduction of amplitude during finger tapping. Time at first visit is the disease duration expressed in months between onset of MND signs and symptoms and first evaluation at our Center. ALS, amyotrophic lateral sclerosis; B, bulbar; CBS, corticobasal syndrome; F, female; LL, lower limbs; M, male; N, no; NA, not available; Park, parkinsonism PD, Parkinson's disease; PLS, Primary lateral sclerosis; PSP, progressive supranuclear palsy; UL, upper limbs; UMNp, upper motor neuron-predominant; Y, yes; y, years*;

*^†^, alive or lost to follow-up*.

Considering all MND patients present in our cohort, extrapyramidal features were significantly more common in PLS than in other MND phenotypes (7/58, 12.1% vs. 11/984, 1.1%; χ^2^ = 38.693, *p* = 5.0x10^−10^). Furthermore, compared to “pure” MND cases, the 18 patients with extrapyramidal features had a significantly older age of MND onset (65.9 ± 10.2 vs. 60.0 ± 12.0; *p* = 0.036), higher frequency of cognitive and/or behavioral impairment (9/18, 50.0% vs. 165/863, 19.1%; χ^2^ = 10.584, *p* = 0.001), of extraocular movement disorders (13/18, 72.2% vs. 80/910, 8.8%; χ^2^ = 78.758, *p* = 7.0 × 10^−19^), and of a family history of neurodegenerative disorders (dementia and/or parkinsonism; 8/18, 44.4% vs. 193/835, 23.1%; χ^2^ = 4.451, *p* = 0.035).

Conversely, no significant differences in sex, survival, site of onset, ALSFRS-R score, progression of disability (delta ALSFRS-R) and MND family history were found.

Genetic screening of major ALS-associated genes was performed in all patients and revealed a p.A383T *TARDBP* mutation in a sporadic ALS-parkinsonism case (ID: 15) and a (G_4_C_2_)_n_ *C9orf72* repeat expansion in a PLS patient with neuropsychological features of CBS and family history of Alzheimer's disease-type dementia (ID: 2) (Supplementary Figure 1). Interestingly, Southern blot on DNA extracted from peripheral blood revealed a large expansion (>3000 repeats), possibly explaining the extrapyramidal and cognitive features observed in our patient. Conversely, genetic screening of major PD-associated genes was performed in 10/18 patients and did not reveal any pathogenic variant (Supplementary Table 1).

## Discussion

In this study we described the clinical characteristics, including genetic testing for major ALS- and PD-associated mutations, in a group of 18 patients with MND and overlapping parkinsonian syndromes. Based on clinical features, we identified two main disease patterns: 1) PLS-parkinsonism and 2) ALS-parkinsonism. PLS patients showed mostly atypical parkinsonian signs, while ALS patients showed mostly overlapping PD (Brait-Fahn disease) (Brait et al., 1973). Among the first group, 6/7 patients met the criteria for a diagnosis of PSP-PLS syndrome (Joseph et al., 2006; Nagao et al., 2012; Höglinger et al., 2017), while the remaining case had a PLS-CBS phenotype (Murakami et al., 2022). The onset of extrapyramidal signs was variable in the different phenotypic groups, often preceding MND diagnosis in ALS-PD patients, while being simultaneous or subsequent in PLS- and ALS-parkinsonism cases. It should also be noted that 3/9 patients with available DaT imaging did not show dopaminergic nigrostriatal degeneration. This is not completely unexpected, since it has been shown that lesions localizing to wide motor networks can cause clinical parkinsonism (Joutsa et al., 2018). Furthermore, as expected levodopa responsiveness was more consistent in ALS-PD cases, while it was documented in only 3/11 participants with MND and atypical parkinsonism. Compared to previous similar studies, our analysis involved a very large, single center cohort of MND patients thus identifying a comparably greater number of patients showing overlapping parkinsonian syndromes (Brait et al., 1973; Qureshi et al., 1996; Mackenzie and Feldman, 2004; Lim et al., 2013). It has been previously reported that up to 69% of ALS patients may show parkinsonian stiffness and 28% may show a parkinsonian syndrome (Pradat et al., 2009; Calvo et al., 2019). However, lower percentages have also been reported, with only 6.8% showing at least two parkinsonian cardinal signs at the time of MND diagnosis (Pupillo et al., 2015). Conflicting percentages are probably due to different study designs and patient characteristics at the time of recruitment. As an example, in a prospective case-control study, the number of participants with certain pre-specified characteristics (e.g. parkinsonian features in MND) may be inadvertently inflated in the recruitment process. Furthermore, MND disease duration at the time of evaluation is also critical, as mild parkinsonian signs may develop in late-stage MND. Therefore, by including only individuals with overt parkinsonian syndromes in our analysis, it is very likely that we missed those patients with very mild symptoms, thus resulting in a lower frequency of MND-parkinsonism cases compared to other reports. On the other hand, however, we can be confident that signs and symptoms detected in our patients, specifically bradykinesia, stiffness and postural instability, are indeed due to a concurrent parkinsonian syndrome, rather than to the underlying MND.

It has been suggested that overlap syndromes involving MND and parkinsonism are more frequent than expected based on population data of the two diseases (Eisen and Calne, 1992). Therefore, it is likely that a shared etiopathogenetic process occurs in these patients, as suggested by the recent observation of a genetic correlation between ALS and PSP (van Rheenen et al., 2016; Chen et al., 2018). Indeed, the finding of an increased frequency of family history for dementia and parkinsonism in our cohort indicates that a common genetic background may exist. Interestingly, if such genetic overlap exists, it is due to mutations in ALS- rather than PD-associated genes. Indeed, two of the 18 patients showed disease-causing mutations in *TARDBP* and *C9orf72*, while we did not observe any variant in genes associated to parkinsonian syndromes. Large *C9orf72* expansions like in our patient are known to be associated with more severe pathological burden in extramotor areas and more complex phenotypes (Brettschneider et al., 2013), while *TARDBP* mutations have been rarely associated to familial PD (Cannas et al., 2013). Notably, in this study the four major ALS-associated genes were systematically tested in MND-parkinsonism patients, whereas genetic analysis has been limited in previous studies.

Neuropathological reports of patients with MND-parkinsonism are conflicting, describing various combinations of Lewy bodies, ubiquitinated cytoplasmic inclusions, tau pathology and atrophy in disease-associated brain regions, as well as a loss of dopaminergic neurons in the substantia nigra (Uitti et al., 1995; Mackenzie and Feldman, 2004; Liang et al., 2005). Since postmortem examination was not performed, we do not have neuropathological information about our patients. However, based on the available clinical, genetic and nuclear medicine data, and on previously mentioned neuropathological studies, we can speculate that patients with ALS and PD may have concurrent TDP-43 and a-synuclein pathology. Conversely, MND-CBS cases may be associated with predominant TDP-43 pathology as suggested by recent reports (Murakami et al., 2022; Seibert et al., 2022), while PLS-PSP cases could be explained by tau, TDP-43, or a combination of both pathologies (Liu et al., 2000).

Limitations of this study include the observational nature of the data with a lack of systematicity in terms of the clinical exams available for each patient (available findings are reported in Table 1 and Supplementary Table 1); the difficulty in distinguishing motor slowing due to UMN impairment from extrapyramidal bradykinesia with decremental amplitude of movements; (Norlinah et al., 2007) the possible random co-occurrence of ALS and PD in the same patient; the unavailability of neuropathological examinations; the lack of systematic screening for other genes responsible for mixed phenotypes, such as *TFG*, (Yoo et al., 2022) or for spinocerebellar ataxias associated with MND and parkinsonian signs, such as *ATXN2*. In the context of MND, the difference between mild spasticity and mild plastic rigidity might not be easily recognizable even by experienced clinicians. Therefore, only the presence of cogwheeling phenomenon was classified as parkinsonian rigidity. Finally, a control cohort was not included in this study. However, the strict inclusion of patients with complex MND-parkinsonian syndromes warrants a comparison with classic MND rather than with elderly healthy controls.

In conclusion, we observed a clear parkinsonian syndrome in 1.7% of patients affected by MND. Although individual phenotypes were heterogeneous, two disease patterns were identified (PLS with atypical parkinsonian syndromes and ALS with typical parkinsonian syndromes resembling PD). Interestingly, these phenotypes may be associated to genetic defects, such as mutations in the *C9orf72* and *TARDBP* genes. It is likely that further investigations into the neuropathological and genetic aspects of these overlap syndromes may yield further insights into the etiopathogenesis of these and other neurodegenerative disorders, and, therefore, on their potential treatments.

## Data Availability Statement

The datasets presented in this study can be found in online repositories. The names of the repository/repositories and accession number(s) can be found below: doi: 10.5281/zenodo.5647409.

## Ethics Statement

The studies involving human participants were reviewed and approved by Istituto Auxologico Italiano IRCCS. The patients/participants provided their written informed consent to participate in this study.

## Author Contributions

JP, FT, and NT: study design and writing of first draft. JP, FT, CM, BP, AC, VS, and NT: data collection. SP, AB, and AR: genetic analysis. JP, FT, FG, and NT: data analysis. NT: supervision. All authors revised the manuscript for intellectual content. All authors contributed to the article and approved the submitted version.

## Funding

This research was supported by the Italian Ministry of Health (GR-2016-02364373 and Ricerca Corrente to Istituto Auxologico Italiano IRCCS – project ‘DAMARE') and AriSLA – Fondazione Italiana di Ricerca per la SLA (Grant AZYGOS 2.0).

## Conflict of Interest

JP is the recipient of a European Academy of Neurology Research Fellowship grant. AR received research funding from AriSLA. VS received compensation for consulting services and/or speaking activities from AveXis, Cytokinetics, Italfarmaco, Liquidweb Srl, and Novartis Pharma AG. He receives or has received research support from the Italian Ministry of Health, AriSLA, and E-Rare Joint Transnational Call. He is on the Editorial Board of Amyotrophic Lateral Sclerosis and Frontotemporal Degeneration, European Neurology, American Journal of Neurodegenerative Diseases, and Frontiers in Neurology. NT received research funding from the Italian Ministry of Health and AriSLA. The remaining authors declare that the research was conducted in the absence of any commercial or financial relationships that could be construed as a potential conflict of interest.

## Publisher's Note

All claims expressed in this article are solely those of the authors and do not necessarily represent those of their affiliated organizations, or those of the publisher, the editors and the reviewers. Any product that may be evaluated in this article, or claim that may be made by its manufacturer, is not guaranteed or endorsed by the publisher.
