# Supplementary material for: Parkinsonian Syndromes in Motor Neuron Disease: A Clinical Study
Source: Front Aging Neurosci. 2022 Jun 27;14:917706. doi: 10.3389/fnagi.2022.917706 (PMC9271964; doi:10.3389/fnagi.2022.917706)
Supplement: Supplementary file 1 [file Data_Sheet_1.PDF]

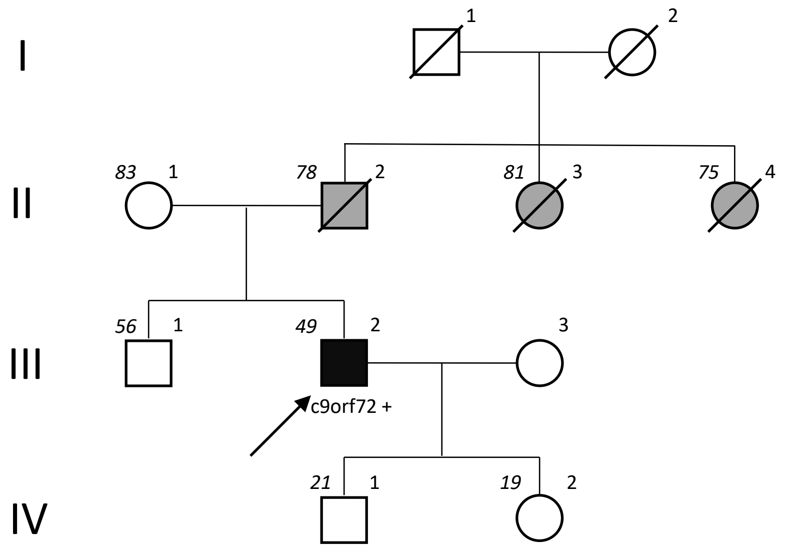

Supplementary Figure 1 – Pedigree of MND-parkinsonism patient ID:2. The arrow indicates the proband. Black: MND. Grey: AD-type dementia. Age at death or at first evaluation of the proband are in italics.
